# Segregation analysis of apolipoprotein A1 levels in families of adolescents: A community-based study in Taiwan

**DOI:** 10.1186/1471-2156-7-4

**Published:** 2006-01-20

**Authors:** Kuo-Liong Chien, Wei J Chen, Hsiu-Ching Hsu, Ta-Chen Su, Ming-Fong Chen, Yuan-Teh Lee

**Affiliations:** 1 Institute of Preventive Medicine, School of Public Health, National Taiwan University, Taipei, Taiwan; 2 Department of Internal Medicine, National Taiwan University Hospital, Taipei, Taiwan; 3 Institute of Epidemiology, College of Public Health, National Taiwan University, Taipei, Taiwan, Department of Psychiatry, National Taiwan University Hospital, Taipei, Taiwan

## Abstract

**Background:**

Apolipoprotein (Apo) A1 is a protective factor for cardiovascular events. This study aimed to perform complex segregation analyses of Apo A1 levels in families of adolescents systematically ascertained from the junior high school students in a rural community. Both siblings and parents of the adolescent probands were recruited for the study. Apo A1 concentrations were measured by turbidimetric immunoassay methods. After adjustment for gender, age, body mass index, smoking and drinking status, residual values of Apo A1 were subjected to subsequent analyses.

**Results:**

Significant mother-father and parent-offspring correlations were found. Commingling analyses indicated that a four-component distribution model was needed to account for the Apo A1 variation. Segregation analysis using regressive models revealed that the best-fit model of Apo A1 was a model of environmental effect plus familial correlation (heritability = 23.9%), in which a significant mother-father correlation existed. Models containing major gene effect could be rejected.

**Conclusion:**

These results suggest that variations of Apo A1 levels in the normal range, especially during adolescence, are likely to be influenced by multiple factors without significant contribution from major genes.

## Background

Apolipoprotein (Apo) A1, one of the structural proteins in high-density lipoprotein particles, is a protective factor against the development of atherosclerotic vascular disease [1,2]. It promotes cholesterol efflux from cells and maintains cellular cholesterol homeostasis. Although the structure of Apo A1 and its corresponding genetic locus have been well characterized [3,4], the levels of Apo A1 are influenced by factors that remain largely unknown. Determinants of Apo A1 concentrations such as gender, age, obesity, and lifestyles, account for only a small proportion of the variance (at most 7%) [5-7]. The DNA polymorphisms of the Apo A1 gene affect Apo A1 concentrations only mildly [8]. Meanwhile, significant genetic contribution to Apo A1 concentrations is indicated by results from family and twin studies [9,10]. The mode of inheritance for Apo A1 concentrations, however, remains to be clarified.

The mode of inheritance as revealed by complex segregation analysis (CSA) can provide evidence whether there is a major gene effect for Apo A1 concentrations, which is important for subsequent gene localization [11]. Even if some major susceptibility genes are already identified, CSA can help shed light on whether other genes exist [12]. So far studies of CSA on Apo A1 concentrations have been conducted mainly in different samples such as from hospital-based patients or community-based adults, with conflicting findings reported [7,8,13-18]. First, earlier CSA in families of probands who either had coronary heart diseases or underwent cardiac catheterization tended to support the existence of major gene effect for Apo A1 concentrations [15,16]. However, a recent study with a large sample in relatives of probands who underwent cardiac angiography failed to find evidence for the major gene effect [8]. Second, two studies in families of adult probands identified from the community found evidence for major gene effect for Apo A1 concentrations [7,17]. But in the HERITAGE Family study, Both Apo A1 at baseline (sedentary state) and its response the training, the major gene was not inferred due to the ambiguous transmission of the major effect from parents to offspring after they followed a supervised exercise training program for 20 weeks [18].

Two reasons for these inconsistent results are possible. First, since coronary heart diseases have a variety of etiologies and indications for angiography examinations vary, ascertaining probands under these clinical conditions would likely lead to etiological heterogeneity in terms of ApoA1 concentrations. Second, gene-environmental interaction may have an important role in the variation of Apo A1 levels and be more prevalent in the adulthood. One way to overcome these limitations is to conduct CSA in families of younger subjects who do not have clinical symptoms and are systematically ascertained. In this study we employed this approach to recruit adolescent probands and their first-degree relatives from a Taiwanese rural community. The study aimed to assess the possible mechanisms of genetic contribution to Apo A1 concentrations through a series of family-genetic analyses, including familial correlation, commingling analysis, and CSA.

## Results

There were 368 probands, 333 siblings and 444 parents in this study. Their distributions on demographic and atherosclerotic risk variables were presented in Table 1. All subjects had average body fatness (mean BMI values of 20 in the probands and siblings, and 24 in the parents). The parents had higher blood pressure, lipid profiles, and smoking and drinking rates than the probands and siblings. In addition, the parents had larger standard deviations in BMI, blood pressure and lipid profiles, especially in triglyceride, than their offspring. The values of skewness and kurtosis showed that Apo A1, Apo B, and triglyceride values were not normally distributed.

**Table 1:** Basic demographic and atherosclerotic risk profiles in this family study, specified by generations (n = 1,145)

	Probands (N = 368)				Siblings (N = 333)				Parents (N = 444)			
			
	Mean	SD	Skewness	Kurtosis	Mean	SD	Skewness	Kurtosis	Mean	SD	Skewness	Kurtosis
Age (years) ***	16.45	0.97	0.33	0.34	17.83	3.43	0.59	1.05	43.65	5.74	1.35	4.65
BW (kg) ***	54.07	12.87	1.04	1.48	52.57	11.03	0.93	2.54	62.87	11.93	1.08	6.02
BH (cm)	161.28	8.46	-0.06	0.03	161.27	8.95	-0.01	1.06	160.90	7.68	0.07	-0.31
BMI (kg/m^2^) ***	20.63	3.87	1.13	1.24	20.08	3.13	1.31	4.26	24.23	3.91	1.03	4.40
SBP (mmHg) ***	108.86	13.02	0.34	-0.34	111.65	12.90	0.36	0.27	119.13	15.12	0.82	1.28
DBP (mmHg) ***	67.79	9.33	-0.05	-0.75	70.66	10.36	0.18	0.51	78.48	11.46	0.70	0.68
TC (mg/dL) ***	168.98	36.43	1.78	11.91	164.94	33.17	0.74	0.70	195.85	35.76	0.29	0.54
TG (mg/dL) ***	85.49	40.14	1.92	7.98	88.86	43.67	2.96	18.43	156.24	134.29	2.66	8.26
HDL-C (mg/dL) *	43.49	10.44	0.50	0.23	44.21	9.55	0.46	0.07	42.26	11.02	0.72	0.77
LDL-C (mg/dL) ***	79.67	32.08	0.61	0.33	86.24	35.61	1.35	4.74	122.13	36.22	0.26	0.61
Apo A1 (mg/dL) ***	108.84	18.19	1.17	4.69	116.49	21.59	1.23	2.81	122.85	23.07	1.06	6.53
Apo B (mg/dL) ***	46.76	13.39	0.35	1.80	49.70	13.87	0.14	0.46	64.53	18.85	1.64	5.32
Smoking ***	1 (0.3%)				35 (10.5%)				159 (35.8%)			
Alcohol drinking ***	1 (0.3%)				25 (7.5%)				173 (39.0%)			

*: p < 0.05, ** p < 0.01, *** p < 0.001 among group difference by ANOVA (for continuous variables) or chi-square test (for categorical variables). Abbreviated: BMI: body mass index, SBP: systolic blood pressure, DBP: diastolic blood pressure, BH: body height, BW: body weight, TC: total cholesterol, TG: triglyceride, HDL-C: high density lipoprotein cholesterol; LDL-C: low density lipoprotein cholesterol, Apo: apolipoprotein.

Proportion of Apo A1 variation in this sample explained by gender, age, age^2^, BMI, smoking and drinking habits was only 7.3%. The correlation between residual Apo A1 concentrations among family members after adjustment for nonlipid covariates is displayed in Table 2. The mother-father correlation coefficient was relatively high as compared with other familial correlations, indicating a strong environment effect and a weak genetic effect on Apo A1 levels. It is possible that there is a sex-specific influence in the variation of Apo A1 levels in the study subjects. The mother-daughter (0.18) and sister-sister (0.23) correlations were higher than father-son (0.11), brother-brother (0.00) or cross-sex correlations for the variation in Apo A1 levels.

**Table 2:** Familial correlation coefficients of residual Apo A1 levels in this family study

		Correlation in Apo A1	
		
	No. of pairs	Equal weight to pairs	Equal weight to pedigrees
Mother-Father	156	0.218	0.218
Parental-Offspring	893	0.122	0.103
Sibling	450	0.119	0.128
Mother-Daughter	240	0.181	0.188
Mother-Son	216	0.107	0.091
Father-Daughter	233	0.087	0.046
Father-Son	204	0.109	0.105
Sister-Sister	136	0.231	0.158
Sister-Brother	221	0.079	0.111
Brother-Brother	93	-0.002	0.037

Commingling analysis showed that a 4-component rather than a single, two or three-component distribution was the best-fit model for Apo A1 variance. The component means, variances, and proportions for the 4-component distribution model were (-0.741, 0.036, 1.142, 2.124), (0.167, 0.593, 4.813, 0.066), and (18.6%, 73.8%, 5.2%, 2.4%), respectively. The χ^2 ^for comparing the 4-component with 3-component distribution was 14.21 (df = 3, p = 0.003), while that for comparing the 4-component with 5-component distribution was 4.31 (df = 3, p = 0.230). The finding of multiple distributions is compatible with a major gene hypothesis; however, commingling may also arise through other causes. Thus, segregation analysis was used to determine whether these major effects segregated in families according to Mendelian expectations.

The results of complex segregation analysis of Apo A1 are presented in Table 3. The sporadic model, familial correlations model, and Mendelian model were rejected due to a *P *value for each model's χ^2 ^of less than 0.001. The environmental model is the best-fit one. Under this model, factor A is pervasively present in the population (frequency = 0.97) and the mother-father correlation coefficient was 0.30. The mother-offspring correlation was higher than the father-offspring correlation (0.26 vs. 0.15). If the four familial correlations in the general model were reduced to two (ρMF = 0.28 ρMO = ρFO = ρSS = 0.18), the fit was not significantly worse (-2lnL = 6332.82, χ^2 ^= 5.50, df = 2, *P *= 0.064). The estimated heritability was 23.9%.

**Table 3:** Parameter estimates from segregation analysis of residual Apo A1 levels under Class D regressive models, conditional on proband phenotypes

Model	Sporadic	Familial correlations	Environmental	Mendelian	General
qA	[1]	[1]	0.974	0.854	0.976
τAA	-	-	=qA	[1]	0.971
τAB	-	-	=qA	[0.5]	0.989
τBB	-	-	=qA	[0]	1.000
AA	118.0	118.4	115.8	118.2	115.9
AB	= AA	= AA	167.2	114.2	167.3
BB	= AA	= AA	287.7	195.9	287.8
σ^2^	492.9	493.2	331.9	390.3	333.3
ρMF	[0]	0.240	0.302	0.267	0.301
ρMO	[0]	0.129	0.263	0.204	0.266
ρFO	[0]	0.081	0.148	0.149	0.148
ρSS	[0]	0.000	0.114	0.088	0.115
Parameter #	2	6	9	9	12
-2ln(L)	6453.22	6435.17	6327.62	6361.51	6327.32
AIC	6457.22	6447.17	6345.62	6370.51	6351.32
χ^2^	125.9	107.85	0.30	34.19	-
*P*	0.000	0.000	0.960	0.000	-

Abbreviation: #, number of parameters; SEM, standard error of mean; qA, gene frequency; τAA, τAB, τBB, transmission probability; μAA, μAB, μBB, genotype means; σ^2^, variance; ρMF, ρMO, ρFO, ρSS, correlation coefficients among spouse, mother-offspring, father-offspring and sibling pairs; #, number of parameters; ln(L), logarithm of likelihood; AIC, Akaike information criteria; *P*, significance level compared with the unrestricted general model.

**Figure 1:**
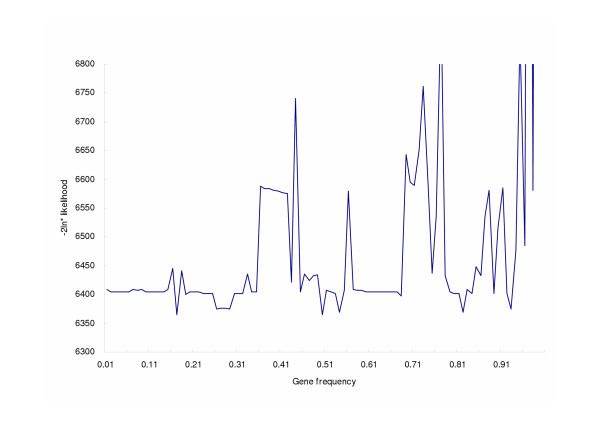
Sensitivity analysis of -2lnL by initial gene frequency values from 0.01 to 0.99, in steps of 0.01, under the Mendelian model of residual Apo A1 levels in this family study.

To rule out the possibility that our rejection of the major gene effect was due to numerical failure, sensitivity analysis of likelihood values was conducted for the Mendelian model. The results are plotted in Figure 1. Only three initial values can reach the global maximum, and the likelihood curves were irregular. The model with parameters estimated under this global maximum could still be rejected as it was compared with the general model.

**Figure 2:**
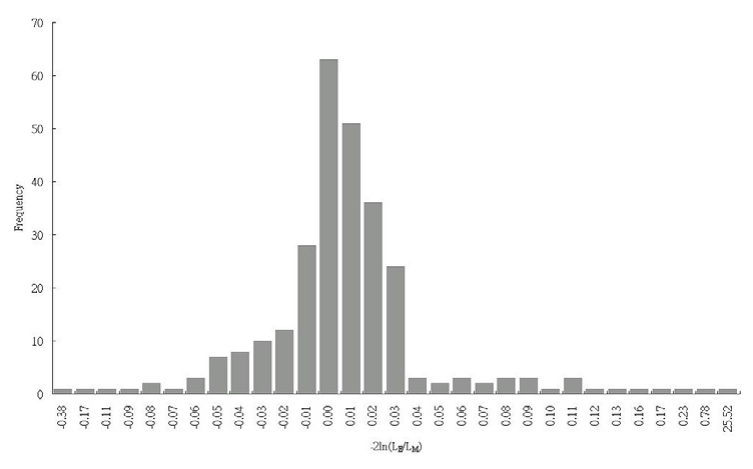
Distribution of families favoring the environmental or Mendelian model in segregation analysis of residual Apo A in this family study, presented by the -2ln(LE/LM) ratio, LE: likelihood of environmental model, LM: likelihood of Mendelian model.

Genetic heterogeneity was tested by plotting -2ln(LE/LM), in which LE refers to the likelihood of the environmental model and LM to that of the Mendelian model (Figure 2). One outlier (i.e., a value of 25.52 for -2ln(LE/LM)) was found favoring the Mendelian model. A repeated segregation analysis after excluding this outlier demonstrated that the environmental model was still the best-fit model.

## Discussion

This study clearly demonstrated that there were significant familial aggregation and commingling components in Apo A1 concentrations among families of adolescents. However, we found that the environmental model that allows for familial correlation rather than the Mendelian model explained the familial aggregation of Apo A1 best.

Two main features of this study are worth noting. First, there is considerable homogeneity in this study population. Most of the subjects live in the same community, hence their social and living environments tended to be more similar than those in different communities. Second, the results are particularly relevant for a population at low risk for atherosclerosis, since the probands were randomly selected from adolescents in the community.

Our results were consistent with those of previous familial correlation and commingling studies [19], which demonstrated that more than one component is needed to explain the distribution of lipids. Previous estimates for the heritability of Apo A1 were 0.2 to 0.3 [20,21]. The high mother-father correlation found in this study indicated that there might be assortative mating and/or common household factors affecting Apo A1 concentrations. We found there were 6.04% parents who knew their hyperlipidemia disease, and less than 1% had history of coronary heart disease. There were only 1.65% parents taking regular hypolipidemic drugs. We think the effects of lipid-lowering drugs were minimal for parent's lipid profiles. We found that the correlation between mother and offspring over Apo A1 was greater than the correlation between father and offspring. Similar results were reported in HDL cholesterol concentrations [22]. Similarity in patterns of lifestyle and physical activity among family members may explain the effects of assortative mating and common household effects [23].

The mode of inheritance of Apo A1 concentrations in this study was best explained by a model of mixed environmental effect and familial correlation. No major gene effect was found. This is similar to the finding of a recent study [18] but contrary to the findings from two studies in families of adult probands identified from the community [7,17]. Several reasons may account for the lack of major gene effect in this study. First, it may reflect the complexity of Apo A1 metabolism. Although Apo A1 is the direct product of a single gene, it is distributed across the full range of HDL particles and other lipoproteins as well. Therefore, Apo A1 concentrations are likely to be influenced by many genetic as well as environmental factors. Hence, this renders the detection of any singular effect of a major gene difficult. Intriguingly, CSA of HDL cholesterol, one phenotype strongly related to Apo A1, also revealed that the environmental model was the best-fit model [24]. Second, there might still be residual confounding on Apo A1 levels from covariates that were not controlled for in this study, such as physical activity and hormone replacement. Third, there were no extreme Apo A1 levels found in this population. Thus, for variation of Apo A1 within the normal range, there might be no major gene effect of considerable magnitude. Finally, the different study population characteristics were one possible reason for incongruent results on the mode of inheritance studies of HDL cholesterol and Apo A1 levels, and this study was presented as subjects with normal range of Apo A1 levels. Also, it is important to consider the inverse relationship between triglyceride and HDL and Apo A1 levels. The rationale for not including triglyceride or HDL in the model was as follows. The genomic profiles controlling Apo A1 and triglyceride or HDL might be overlapped. Adjusting triglyceride or HDL will eliminate the genetic proportions of controlling HDL and triglyceride, and a part of Apo A1 levels. It was contradictory to our primary hypothesis to explore the genetic components of Apo A1 levels. Therefore, the genetic results may be confounded by triglyceride or HDL components, and did not separate the background of specific traits.

Another possible reason for our failure to detect a major gene effect for Apo A1 is the young age of our subjects. Age plays an important role for genetic control on phenotype expression, and environmental factors add complication in lipid traits. A recent study on the effect of quantitative trait loci for lipid phenotypes in the rat indicated that genetic components become important factors to the control of phenotype expression as age increases [25]. Among human, Apo A1 was also reported to have intergenerational differences in heritability [26]. However, lack of a major gene in the study families does not exclude the possibility that there are major genes that will influence Apo A1 levels in late adulthood. For example, there have been reports suggesting pleiotropy affecting lipid-related traits and obesity. Substantial evidence for quantitative trait loci with pleiotropic effects influencing BMI and HDL were found in the Framingham Heart Study [40]. We conducted analyses with BMI as a covariate. If pleiotropy exists affecting body weight and lipid-related traits, adjusting for BMI would likely hide the genetic effect on ApoA1. This could explain why our study did not identify a major gene contribution. Finally, we have collected several items about food intakes among the family members, such as vegetarian, meats, and rice amounts. The high spouse correlation coefficient was not associated with above variables. It is the limitation of our study to explain the possible common household effects on Apo A1 levels.

Segregation analysis is typically very sensitive to ascertainment, and false assumptions on ascertainment could invalidate the estimates obtained through segregation analysis (11). Highly selected samples obtained through a phenotype-based ascertainment scheme may lead to biased estimates. Therefore, the ascertainment scheme is very important on segregation analysis. Although we did not ascertain probands on ApoA1 trait, the likelihoods corrected for ascertainment on probands' ApoA1 levels provided efficient estimation. The results of likelihood without correction of proband ascertainment were similar to corrected likelihood results.

Complex segregation analysis was used to ask whether an inheritable trait is controlled by a single major gene plus residual polygenes, if so, gene mapping tasks would be warranted. If not, gene mapping studies would be lack of power because traditional statistical programs and sparse genetic markers would not provide sufficient tools to tackle a trait without major gene effects. However, this situation has changed in the past few years. Now we know a complex trait is less likely to be influenced by a single major gene. Advanced statistical programs, dense genetic markers plus other new technologies has allowed us to investigate a complex trait where genes with small to moderate effects. In the post-genomics era, segregation analysis is considered as an intermediate tool to help investigators to plan further sophisticated genomic studies. Although the method did not give information of exact DNA locus to find the genes, it can provide heritability estimates and parameters for further parametric linkage analysis. So segregation analysis should be useful and not obsolete. Further genotype-based methods such as linkage and association will be planned for elucidating genetic effects and locations.

The large proportion of un-response parents was the limitation of the study. Only 60% parents attended the study. The causes of low response rates were as follows. First, the parents were in the productive age (mean 44 years old) and they felt they were relatively healthy and were not interested in the health checkup. Second, the medical history rates of atherosclerotic disease among participant parents were very low. We found there were 6% participant parents who knew their hyperlipidemia disease, and less than 1% had history of coronary heart disease. Although we cannot collect the data on un-response parents, we have checked the questionnaires of lifestyle patterns from the probands, and found the distributions of socioeconomic status and lifestyle patterns were similar between response and non-response parents. It might imply the response parents can be the representatives of all parents.

## Conclusion

Variations of Apo A1 levels in the normal range, especially during adolescence, are likely to be influenced by multiple factors without significant contribution from major genes.

## Methods

### Subjects

This family study was part of the Chin-San Community Cardiovascular Study, a prospective cohort study began in 1990 [27,28]. The family study arm started in 1997 and was designed to recruit adolescent probands from students in the only junior high school in the community. At first, a total of 1063 students (response rate 94.6%) agreed to participate in a general health check-up after informed consent was obtained. They underwent examinations for anthropometric measures, blood pressure, and lipid profiles, including total cholesterol, triglyceride, low density lipoprotein (LDL) cholesterol, body mass index, systolic pressure, diastolic pressure, and high density lipoprotein (HDL) cholesterol. The high-risk young probands were defined as the highest scores of above seven measures, which were 90^th ^percentile values among all subjects (HDL as below 10^th ^percentile, n = 171). The control young probands were ascertained by random sampling of other young students (n = 197). Because the original stratification was not based exclusively on the Apo A1 and the results of CSA for the families of the two strata were similar, only the results of all families together with correction for ascertainment on proband's Apo A1 were reported in this study. After obtaining informed consent from probands' family members, the same measures were performed for each family member. Only first-degree relatives were included for this genetic study, with a total of 1,145 subjects.

### Measurement of lipid profiles

The measurement of various lipid profiles have been described in detail elsewhere [29]. Briefly, a blood sample of 20 ml was drawn from each participant after a 12-hour overnight fast. Enzymatic methods were used to determine serum cholesterol and triglyceride (Merck 14354 and 14366 Germany, respectively). The HDL-C level was measured in the supernatant after precipating with magnesium chloride phosphotungstate reagents (Merck 14993, Germany). The LDL-C content was measured as "total cholesterol minus cholesterol in the supernatant" by the precipitation method [30]. (Merck 14992, Germany). If the triglyceride concentration was more than 400 mg/dL, cholesterol was measured from an infranate density more than 1.006 from ultracentrifugation [31]. Apo A1 and B concentrations were measured by turbidimetric immunoassay using commercial kits (Sigma, USA). The standard samples for apolipoprotein assay were from the Center for Disease Control, USA. The coefficient of variation of Apo A1 measured in our laboratory was less than 3%. If extreme values were found, repeated measures were done for confirmation. All measures were performed in July and August, and hence seasonal variation could be minimized.

### Statistical analyses

We adjusted a person's Apo A1 concentrations for known determinants by regressing for age, gender, body mass index (BMI), smoking and alcohol drinking status. Both age and BMI were centered by subtracting the sample mean, 34.0 for age and 22.3 for BMI, from the corresponding variables. Residual values plus the sample mean of Apo A1 (116.8 mg/dL) were then used for further analyses.

The correlations of Apo A1 concentrations between mother-father, parent-offspring, and siblings were estimated by using the FCOR program in SAGE [32]. Next, commingling analysis of Apo A1 levels was performed using the ADMIX program to test whether the data were best described by one, two, or more component distributions [33]. The parameters for each component's mean, variance, and proportion were estimated by the maximum likelihood method. Hypothesis testing for nested models was carried out with the likelihood ratio test.

### Complex segregation analysis

Segregation analysis of Apo A1 levels was conducted using regressive models as implemented in the REGC program in SAGE These models assume that variation of Apo A1 concentration among family members is the result of a major gene effect, with the residual variation reflecting both familial correlations and individual variation. The presence of a major gene is assessed by allowing two factors or alleles (A and B) at a single locus, resulting in three 'ousiotypes' (AA, AB, BB) in individuals (Cannings et al. 1977). The means of Apo A1 for each ousiotype is denoted AA, AB, BB, with one common variance of σ^2^. The frequencies of allele A and B are denoted qA and (1-qA), respectively. The distribution of types in the population is assumed to be in Hardy-Weinberg equilibrium. Individuals of each type are assumed to transmit allele A to their offspring with transmission probabilities τAA, τAB and τBB, respectively. Residual familial resemblance not explained by this major locus is modeled by familial correlations. The correlation between spouses, parent and offspring, mother and offspring, father and offspring, and between siblings, are denoted ρMF, ρPO, ρMO, ρFO, and ρSS, respectively. For this study, we adopted class D regressive models, in which residual sib-sib correlations are equal among all sibs of common parentage and can be due to any cause. If ρPO is held equal to ρSS, these models have been shown to be mathematically and numerically equivalent to the conventional mixed model of inheritance in nuclear families [34]. Under this circumstance, the heritability can be estimated by 2ρSS(σ^2^/σ^2^T), where σ^2^T is the total variance, and σ^2 ^is the variance conditional on the major ouisotype [34].

The analyses started with fitting a general model, in which all parameters were allowed to be estimated. Then we compared the general model with various submodels in which certain parameters were restricted to specific values. Under a Mendelian model, the transmission probabilities, i.e., τAA, τAB and τBB, were held equal to Mendelian expectations of 1, 0.5, and 0. A nontransmitted environmental effect model predicts that the probability that an individual has one ousiotype or another is independent of both the person's generation and the ousiotypes of his/her parents. For the environmental model in this study, each of the transmission probabilities is taken to be equal to the factor frequency, i.e., τAA = τAB = τBB = qA. Both the Mendelian and environmental models can allow for residual familial correlations. A pure polygenic model assumes no major gene effect, so gene frequency and transmission probabilities are all fixed to one. The fit of hierarchical models is compared with the likelihood ratio test, calculated as -2 of the difference between the *ln *likelihood of the models being compared. The likelihood ratio value follows a chi-square distribution, with degrees of freedom equal to the difference between the models in the number of parameters estimated. Among nonhierarchical models, the most parsimonious model is that with the lowest values of Akaike's information criterion (AIC = -2 ln likelihood + 2 [number of estimated parameters]) [35].

We used the adjusted Apo A1 values without logarithm transformation for the segregation analysis because normalizing transformation of a biologically skewed trait would decrease power to detect a major gene effect when one exists [36]. Instead, we fit the environmental model. If such a model can be rejected, the major gene effect will not be due to skewness of the residual Apo A1 levels [37]. Ascertainment correction was conducted by conditioning on the phenotypes of probands.

A sensitivity test of the model fitting, specified by initial values of gene frequency, was performed to differentiate local maxima from global maximum [38]. We calculated the -2lnL values by setting initial gene frequency to values ranging from 0.01 to 0.99, in steps of 0.01, and fixed initial means, variance, and familial correlations. We compared likelihood values of two models and calculated the ratio of log likelihood values to detect possible heterogeneity [7,39].

## Authors' contributions

KLC carried out the data collection, statistical analyses, participated in the study design and processing data. WCC & YTL participated in the design of the study and supervised the ideas developing in hypothesis generation. MFC & HCH performed the laboratory measurement in lipid levels and in charge of quality control. All authors read and approved the final manuscript.
